# Metabolic adjustments in neonatal dwarf and normal-sized goat kids: Relationship between serum metabolites and body size

**DOI:** 10.1371/journal.pone.0289809

**Published:** 2023-11-16

**Authors:** Buhari Habibu, Tagang Aluwong, Lukuman Surakat Yaqub, Hajarah Uwale Buhari, Hussaina Joan Makun, Mohammed Umaru Kawu

**Affiliations:** 1 Department of Veterinary Physiology, Ahmadu Bello University, Zaria, Nigeria; 2 Samaru College of Agriculture, Division of Agricultural Colleges, Ahmadu Bello University, Zaria, Nigeria; 3 National Animal Production Research Institute, Ahmadu Bello University, Zaria, Nigeria; Ain Shams University Faculty of Agriculture, EGYPT

## Abstract

The relationship between body size and metabolism of goats remains poorly studied. The study evaluated the neonatal metabolic adjustments and elucidated the relationship between serum metabolites and body size in 39 single-born dwarf and normal-sized goat kids. Body weight, length and height of kids were recorded at birth and blood samples were collected from the dwarf and normal-sized (Red Sokoto and Sahel) goats on Days 0 (birth), 3, 10 and 20, postnatal. Also, the body mass index (BMI) was calculated and the concentration of metabolic markers was determined. Results revealed that values of BMI, body weight, length and height were lowest (P < 0.01) in the dwarf, followed by values in Red Sokoto kids, while the Sahel kids had the highest (P < 0.01) values. Conversely, the concentration of triglyceride at birth was highest (P < 0.05) in the dwarf, moderate in Red Sokoto and lowest in Sahel goats. Similarly, the Sahel goat kids had the lowest neonatal (P < 0.05) concentration of serum cholesterol. Neonatal concentrations of serum albumin and urea were higher in Sahel than Red Sokoto (P < 0.05) and the dwarf (P > 0.05) goats. Concentration of serum albumin was lower (P < 0.05) at birth, but significantly increased later, while values of serum urea concentration were higher (P < 0.05) at birth, but significantly decreased in subsequent postnatal days. Unlike the BMI, birth weight showed significant negative (P < 0.05) correlation with the concentration of most serum metabolites, especially triglyceride, which showed negative correlation at birth and in subsequent postnatal days. We concluded that dwarfism or small body size is associated with high serum triglyceride in single-born neonatal goats, and this is probably due to the accumulation of body energy reserve in the form of body fat to compensate for lower body tissue mass.

## Introduction

Domestic goats are subdivided into three categories based on body size, namely: dwarf, small and large breeds [1]. Caprine dwarfism seems to have originated from Africa, thus all dwarf goats of the world are primary descendants of two African breeds, namely: West African Dwarf (WAD) and South Sudan Dwarf breeds [2]. The West Africa Dwarf goats are the most numerous and well spread; they are indigenous to several countries in West and Central Africa. The WAD, Red Sokoto and Sahel breeds of goat are indigenous to most West African countries and play an imminent economic role in the livestock sector of the sub-continent [3].

Neonatal goats seem anatomically complete at the kidding point, but are physiologically immature; needing maturation in metabolism, immunity and thermoregulation [4]. Biomarkers of metabolism, including serum lipids and proteins; undergo significant changes in their concentrations during neonatal life, as tissues, especially the liver, mature with advancing age [4, 5]. After birth, several physiological adjustments help neonates to maintain an independent metabolic homeostasis [6]. Heavier neonates are better able to improve metabolism, due to enhanced brown fat metabolism and non-shivering thermogenesis [7, 8]. Breeds that better resist cooling during neonatal life have a body weight advantage and more efficiently mobilize stored energy from brown adipose tissue to support neonatal activities [8].

Lipid homeostasis in higher organisms is controlled via an integrated system capable of prompt response to metabolic changes [9]. Triglycerides (triacylglycerol) are esters derived from glycerol and three fatty acids and are the main constituents of body fat [10]. They are present in the blood to enable the bidirectional conveyance of adipose fat and blood glucose from the liver [10, 11]. Since excess calories are stored in the form of triglycerides in adipocytes and released under the influence of glucagon, to be catabolized in all body tissue (except the brain) to produce energy between meals [11]. Blood triglycerides serve as sources of energy fuel and are hydrolyzed to release free fatty acids and glycerol [6, 9]. Cholesterol plays an essential role in stabilizing the architecture of the plasma membrane, serves as a precursor for bile acid and steroid hormone synthesis, and participates in growth, replication and maintenance in mammals [12]. Changes in total cholesterol concentration reflect changes in the various lipoprotein fractions and their sub-components during the neonatal life of goats [6]. Urea serves as a means of excreting nitrogen, obtained mainly from the metabolism of amino acids [13, 14]. Higher serum urea levels may indicate an excess of protein ingestion, increased use of the body amino acids stores to provide metabolizable energy or a carbohydrate deficiency, as proteins are catabolized in order to spare glucose oxidation [15, 16].

In rodents and humans, dwarfism is associated with growth hormone deficiency and obesity [17, 18]. The birth weight of animals is well known to significantly influence neonatal metabolic response [7, 8]. In humans, an increase in birth weight is associated with decreased blood triglyceride concentration, such that a 1 kg increase in birth weight is accompanied by a 10% decrease in blood triglyceride concentration [19, 20]. A reasonably high birth weight is of uttermost importance to the survival of neonates. High birth weight may imply large energy reserves to support neonatal metabolic adjustment. Thus, information on birth weight could be useful in predicting the expected metabolic adjustments in neonatal tropical goat kids, and may serve as a valuable tool in selective feed based on birth weight, and manipulation of maternal and neonatal diet to improve metabolic adjustments and enhance neonatal survival [8]. Poor understanding of neonatal metabolic maturation in breeds of goat with different body size, due to paucity of information may, in part, contribute to the high neonatal mortality in West African goats [4, 21]. We hypothesized that no difference exists in neonatal metabolic adjustments in goats, irrespective of body size. Thus, the aim of the current study was to evaluate the neonatal metabolic adjustments in dwarf and normal-size breeds of tropical goat kids, and also elucidated the relationship between serum metabolites and morphometric variables in single-born kids.

## Materials and methods

### Experimental location and animal management

The adopted study protocol and experimental design followed the international guidelines for animal welfare and got the approval of the Animal Use and Welfare Committee of Ahmadu Bello University: ABUCAUC/2019/13. Efforts were made to reduce suffering of the kids during the experiment; no kid was sacrificed or anesthetized. The animals used for the study were housed in the National Animal Production Research Institute (NAPRI), Ahmadu Bello University, Shika, Zaria, Nigeria (latitudes 11 and 12° N and longitudes 7 and 8° E). Each well-ventilated pen had a maximum stocking density of 20 does and dimensions of 6.1, 6.1 and 2.2 m for length, width and height, respectively.

More than 200 does with parity ranging between 1 and 2 were used for the study. The does in three breeds were balanced for parity. They were synchronized by injecting 1 mL of cloprostenol (Synchromate^®^; half the manufacturer’s recommended dose for a cow) into the thigh muscle. Does that showed signs of oestrus 24 h post-treatment, were housed in separate pens of 5 does per pen and a proven buck was introduced to each pen.

In all does, the kidding was vaginal without assistance and the kids had no apparent congenital abnormalities. The litter-size of the dams and gender of the kids were recorded immediately after parturition. The kids were allowed to suckle their dams directly. Basic clinical examination was carried out on each neonate after parturition, emphasising more on the presence of good sucking reflex and absence of asphyxiation or foetal-fluid aspiration during kidding. The dams grazed on natural pasture during the day, and supplemented with *Digitaria smutsii* hay and concentrate ration at 3% body weight daily. Water was provided *ad libitum*. Management of the goats followed standard goat husbandry protocols.

### Breeding of goats and study design

Only single-born kids (n = 32) were used for the study. A total of 39 single-born kids, belonging to Red Sokoto (n = 12), Sahel (n = 12) and WAD (n = 12) goats were used for the study. Eighteen (n = 17) of the kids were buck-kids and 19 were doelings. After commencement of suckling, the kids were allowed with their dams for 10 minutes to strengthen maternal-kid bonding. Then, they were restrained gently by experienced personnel, using a gloved-hand to collect blood samples on the day of birth. Subsequently, blood sample collection was repeated on Days 3, 10 and 20, postnatal. Blood samples were collected in the morning (08:00 h GMT + 1) by jugular veni-puncture. The birth weight and morphometric variables were determined using a weighing scale (Salter, Model 250, England) and measuring tape. Body length was measured as the length from the external occipital protuberance to the base of the tail and body height was measured as the length from the surface of a platform to the withers [22].

### Determination of metabolite concentrations

The blood samples were gently transferred into anticoagulant-free tubes and allowed to clot, followed by centrifugation at 3,000 revolutions per minute for 15 minutes. The serum was decanted into serum sample bottles, capped and stored at -20°C until analysed for serum total proteins, albumin, urea, triglyceride and cholesterol. Using Randox^®^ reagents, spectrophotometer was used to determine the concentration of serum total proteins, albumin, urea [23], triglyceride [24] and cholesterol [25]. The globulin value was calculated by subtracting the value of albumin from that of total protein. Serum concentration of immunoglobulins was determined using the zinc turbidity test as first described by McEwan et al. [26] and recently modified by Hogan et al. [27].

### Data analysis

The Statistical Package for Social Science (SPSS) version 21 was used for the analysis. Descriptive statistics was performed on all variables and values were expressed as mean ± standard error of the mean. The effects of age, breed and sex on concentration of serum metabolites were evaluated for significance using repeated measures analysis of variance (ANOVA) in a general linear model (GLM) with Greenhouse–Geisser correction. Effect of breed on BMI, body weight, length and height were evaluated for significance using one-way ANOVA. Multiple comparison of experimental groups was performed using Tukey honestly significant difference (HDS) test to separate the means. Interactions between experimental groups and time (age) were assessed to evaluate the effect of advancing age in the neonates. Pearson’s correlation was used to compare the relationship between values of serum metabolites and birth weight or BMI. Discriminant analysis was performed to identify variables with greater discriminatory power between the three breeds and was followed by discriminant analysis option of variable classification. Boxplot was constructed to show the centrality, spread and symmetry of the outcome variables in each experimental group. Comparisons in all cases were done at the 5% level of significance.

## Results

### Variables indicating body size

**Fig 1:**
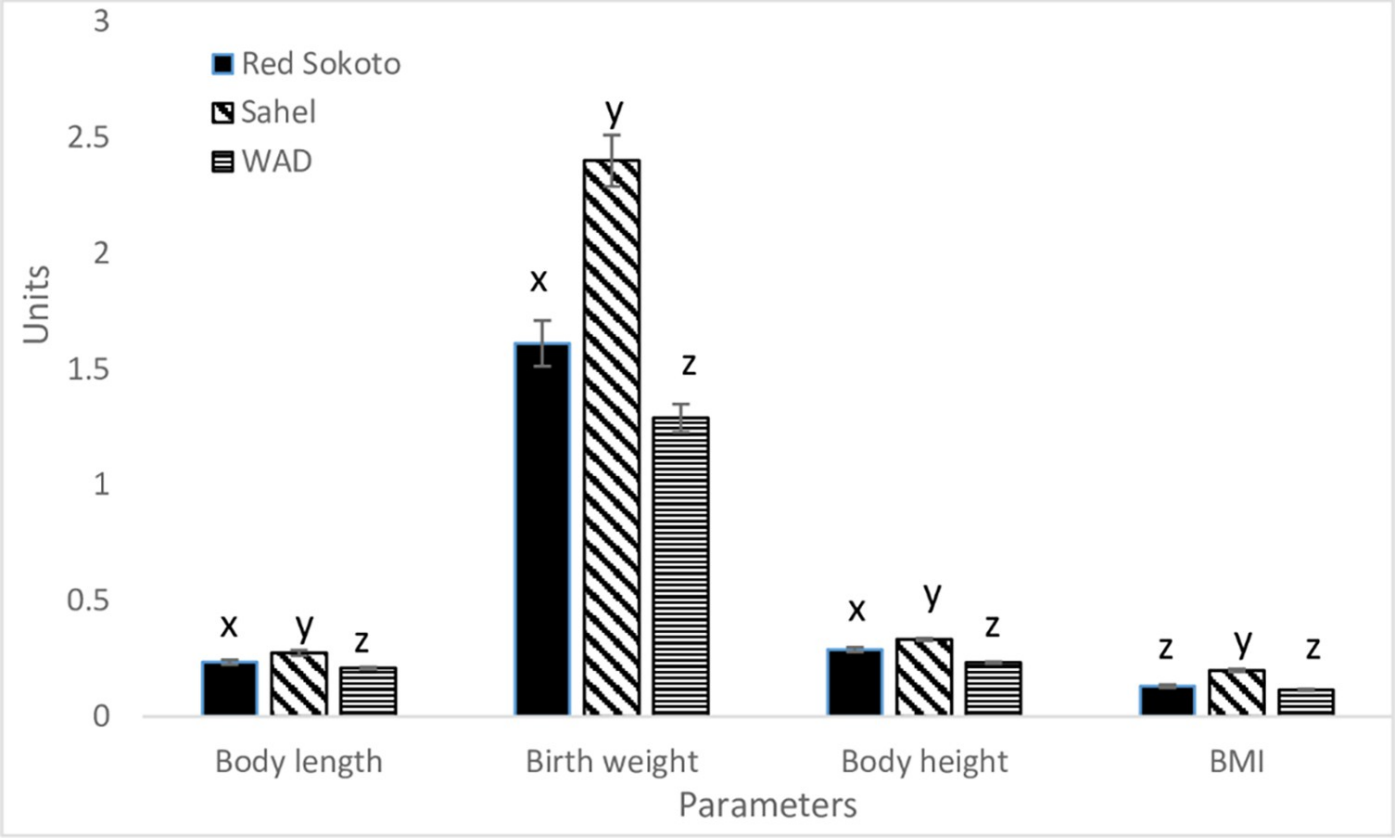
Effect of breed on body length (m), weight (kg) height (m) and BMI (body mass index) in Red Sokoto, Sahel and WAD (West African Dwarf) goat kids. Values (mean ± Standard error) with x, y, z indicate significant difference (P < 0.05) between breeds.

The mean values of variables showing body size were presented in Fig 1. The dwarf kids had the lowest (P < 0.01) mean values of body weight, length and height at birth, while the Sahel goats had the highest (P < 0.01) mean values. The kids of Red Sokoto goats had higher mean values than the dwarf kids, but lower mean values than the Sahel kids. Similarly, BMI was higher (P < 0.01) in Sahel than Red Sokoto and dwarf goats, whereas the breed difference was not significant (P > 0.05) between Red Sokoto and dwarf goats.

### Neonatal metabolic adjustment

**Fig 2:**
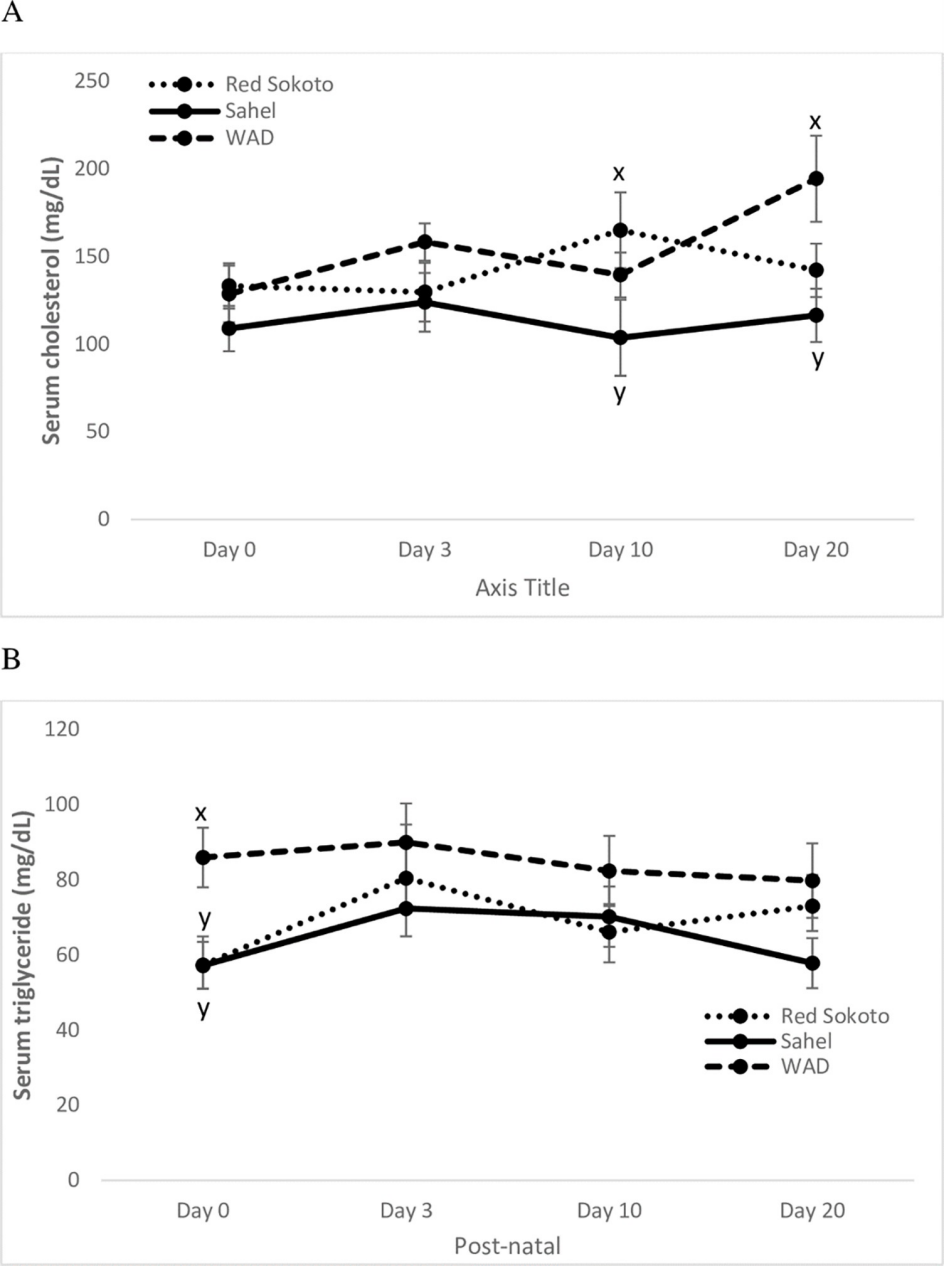
Postnatal changes in serum lipid profiles (cholesterol; A and triglyceride; B) in Red Sokoto, Sahel and WAD (West African Dwarf) goats. Values (mean ± Standard error) with x, y indicate significant difference (P < 0.05) between breeds. Time x breed interaction was significant for cholesterol (P < 0.01).

**Fig 3:**
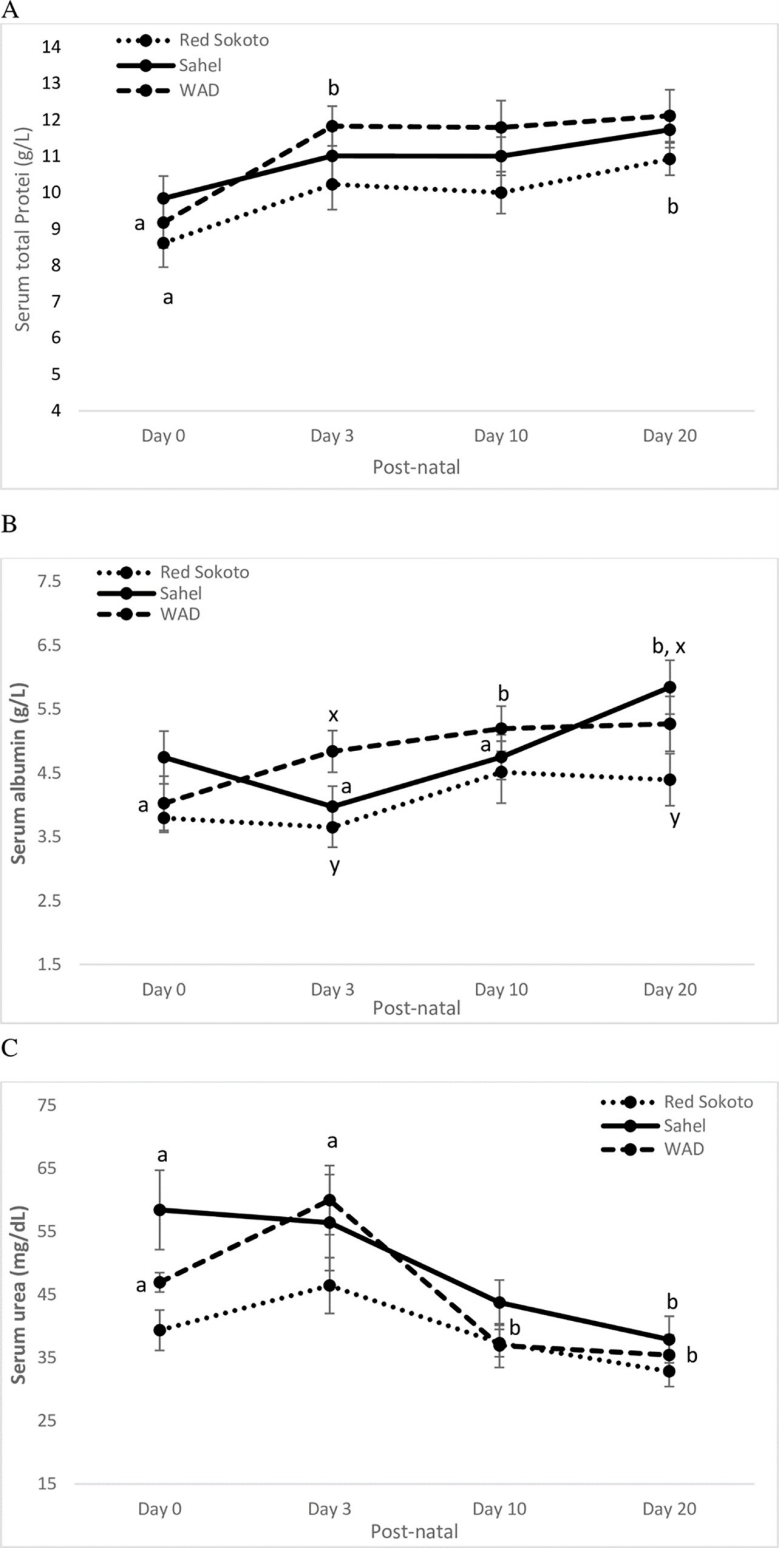
Postnatal changes in serum protein profiles (total proteins; A and albumin; B) and urea (C) in Red Sokoto, Sahel and WAD (West African Dwarf) goats. Values (mean ± Standard error) with x, y indicate significant difference (P < 0.05) between breeds. Time x breed interaction was significant for albumin (P < 0.01).

**Fig 4:**
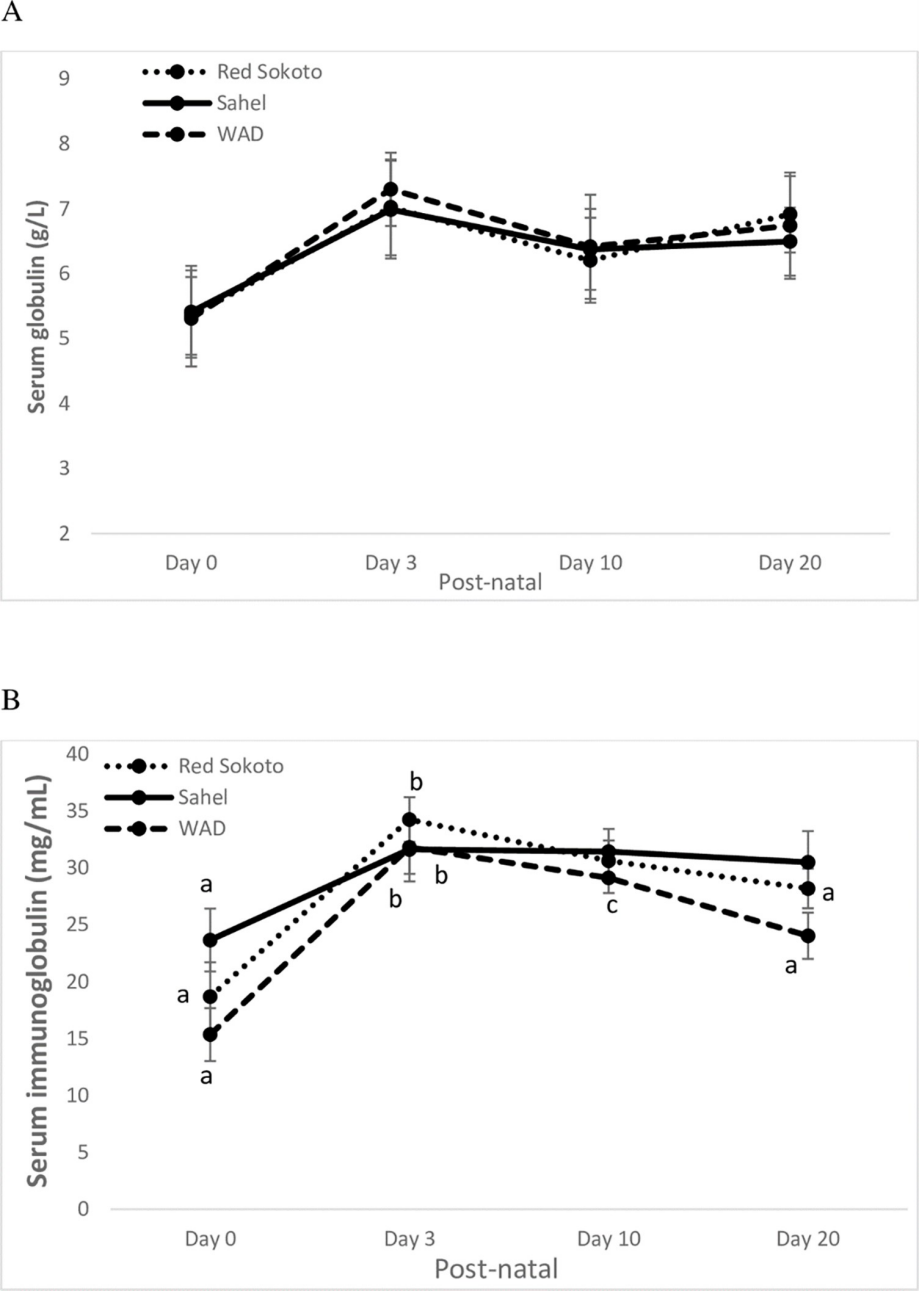
Postnatal changes in immunity profiles (globulins; A and immunoglobulins; B) in Red Sokoto, Sahel and WAD (West African Dwarf) goats. Values (mean ± Standard error) with a, b indicate significant difference (P < 0.05) between postnatal days.

**Fig 5:**
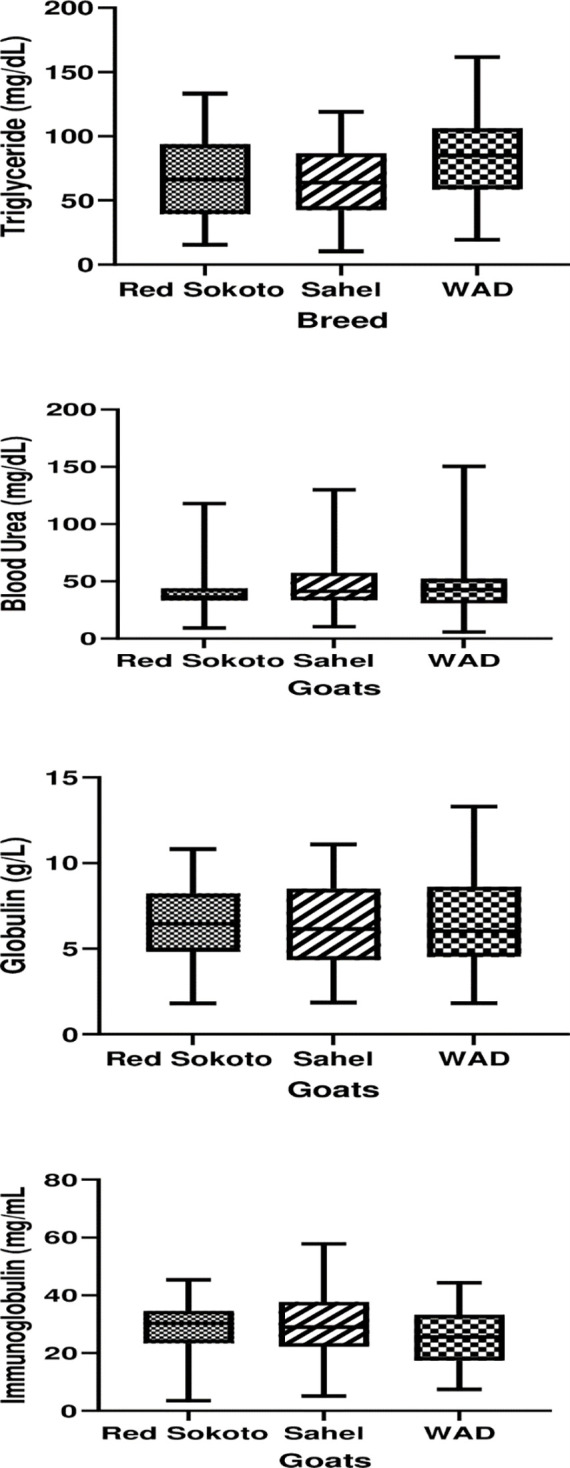
Boxplots of birth weight and serum metabolites in Red Sokoto, Sahel and WAD (West African Dwarf) goat kids during neonatal period.

Age-dependent changes in metabolic parameters in the dwarf and normal-size breeds of goats during neonatal life is presented in Figs 2–5, while the pooled data are shown on Table 2. Serum total cholesterol (Fig 2A; Age: P > 0.05; Breed: P < 0.05; Interaction: P < 0.05) and triglyceride (Fig 2B; Age: P > 0.05; Breed: P < 0.05; Interaction: P > 0.05) in the three breeds showed no significant fluctuation from birth to Day 20. During late neonatal life and in the overall data, Sahel goat kids had significantly (P < 0.05) lower serum cholesterol than Red Sokoto and dwarf goat kids. Whereas, dwarf goat kids had higher serum triglyceride than the normal-size breeds on the day of birth and in the overall data.

The values of serum protein profiles (Fig 3) showed that serum total proteins (Fig 3A Age: P < 0.05; Breed: P < 0.05; Interaction: P > 0.05) were lower at birth in the dwarf and Red Sokoto goat kids, but significantly (P < 0.05) increased in early and late postnatal life, respectively. Values of serum total proteins were relatively higher at birth in Sahel goat kids, but no significant (P > 0.05) change was observed throughout the neonatal period. The neonatal Red Sokoto goats had lower (P < 0.05) serum total proteins than dwarf goats in the overall data. On the other hand, values of serum albumin concentration (Fig 3B; Age: P < 0.05; Breed: P < 0.05; Interaction: P < 0.05) were lower at birth, but significantly (P < 0.05) increased subsequently in dwarf and Sahel goat kids. However, values of serum albumin concentration were lower in Red Sokoto goat kids as compared with the other breeds and the kids of Red Sokoto goats maintained a lower value with no significant (P > 0.05) fluctuation from birth to Day 20. Conversely, both Sahel and dwarf kids had higher serum urea (Fig 3C; Age: P < 0.05; Breed: P < 0.05; Interaction: P > 0.05) at birth, which significantly (P < 0.05) decreased subsequently. Similarly, neonatal Red Sokoto goats maintained a lower value of serum urea with no significant (P > 0.05) fluctuation from birth to Day 20. In the pooled data (Table 1) and on Days 3 and 20 (Fig 4), both serum albumin and urea were significantly (P < 0.05) lower in Red Sokoto than Sahel and dwarf goat kids.

**Table 1:** Influence of breed on birth weight and serum metabolites in Red Sokoto, Sahel and WAD (West African Dwarf) goat kids during neonatal period.

Metabolites	Red Sokoto	Sahel	WAD	SEM	P value
Total cholesterol (mg/dL)	142.50 a	113.20 b	155.20 a	7.86	0.007
Triglycerides (mg/dL)	69.16 a	64.35 a	84.44 b	4.35	0.0015
Total proteins (g/L)	10.02 a	10.89	11.30 b	0.33	0.019
Albumin (g/L)	4.087 a	4.828 b	4.78 b	0.20	0.013
Blood urea (mg/dL)	39.39 a	49.13 b	46.81	3.07	0.027
Globulins (g/L)	6.37	6.312	6.44	0.34	0.55
Immunoglobulins (mg/mL)	27.95	29.31	25.08	2.95	0.12

Values with a, b indicate significant (P < 0.05) breed difference.

Kids of the three breeds had a significant increase (P < 0.05) in concentration of circulating immunoglobulins (Fig 4B; Age: P < 0.05; Breed: P > 0.05; Interaction: P > 0.05) after birth (Day 0 vs Day 3). Subsequently, a significant decrease in concentration of circulating immunoglobulins was observed in Red Sokoto kids during late neonatal life (Day 3 vs Day 20), while the decrease in the dwarf kids was markedly (P < 0.05) progressive (Day 3 vs Day 10 vs Day 20). In Sahel, however, no significant (P > 0.05) fluctuation was observed in circulating concentration of immunoglobulins subsequently. Age and breed had no effect on serum concentration of globulins throughout the course of the study (Fig 4A and Table 1).

### Association between body size and serum metabolites

The relationship between serum metabolites and both body weight and BMI at birth are presented on Tables 2 and 3, respectively. A significantly negative (P < 0.05) correlation was observed between birth weight and triglyceride concentration in early and late postnatal life. Also, significantly negative (P < 0.05) correlation was observed between birth weight and cholesterol on Day 10. Of all the metabolic parameters, only cholesterol showed significant correlation with BMI on Day20 (Table 3). Serum urea concentrations showed significantly negative (P < 0.05; r = -0.54*) correlation with birth weight in early neonatal life (Day 3; Table 2). Circulating immunoglobulin concentrations showed significantly positive (P < 0.05; r = 0.36) correlation with birth weight in late neonatal life (Day 20; Table 2).

**Table 2:** Pearson’s correlation between birth weight and serum metabolites in Red Sokoto, Sahel and WAD (West African Dwarf) goat kids during neonatal period.

Metabolites	Birth weight (kg)			
	Day 0	Day 3	Day 10	Day 20
Total cholesterol (mg/dL)	-0.22	-0.16	-0.36*	-0.23
Triglycerides (mg/dL)	-0.40*	-0.38*	0.06	-0.37*
Total proteins (g/L)	0.24	-0.16	-0.01	0.14
Albumin (g/L)	0.14	-0.27	-0.03	0.131
Blood urea (mg/dL)	-0.02	-0.54*	-0.02	-0.03
Globulins (g/L)	0.10	-0.07	-0.03	0.08
Immunoglobulins (mg/mL)	0.22	0.09	0.17	0.36*

Values with asterisk (*) indicates significant correlation at P<0.05

**Table 3:** Pearson’s correlation between body mass index (BMI) and serum metabolites in Red Sokoto, Sahel and WAD (West African Dwarf) goat kids during neonatal period.

Metabolites	Goat body mass index (gBMI)			
	Day 0	Day 3	Day 10	Day 20
Total cholesterol (mg/dL)	-0.24	-0.24	-0.34	-0.43 *
Triglycerides (mg/dL)	-0.30	-0.28	0.09	-0.21
Total proteins (g/L)	0.19	-0.07	0.16	0.20
Albumin (g/L)	0.08	-0.24	-0.10	0.16
Blood urea (mg/dL)	-0.03	-0.34	-0.03	0.10
Globulins (g/L)	0.15	0.04	0.08	0.08
Immunoglobulins (mg/mL)	0.19	-0.06	0.15	0.12

Values with asterisk (*) indicates significant correlation at P<0.05

### Pattern of metabolites in each breed and sexual dimorphism

Generally, the boxplot (Fig 5) shows that Sahel goat kids seem reasonably more symmetrical in the distribution of most metabolic parameters than the other breeds. Also, the variability of WAD goat kids was larger for all metabolic parameters, except for immunoglobulin concentration. Specifically, serum urea was skewed to the right in the three breeds. The discriminant function classification shows similarity in the metabolic response of the three breeds during the early and late neonatal life (Table 4). Nonetheless, the misclassification was higher during late neonatal life. Serum concentration of cholesterol and triglyceride mostly accounted for the variations in the adjustments of the three tropical breeds to neonatal life (Table 5). Sexual dimorphism was only observed in concentrations of albumin and globulin; doelings had significantly (P < 0.05) higher serum albumin concentration than buck-kids, while serum globulin concentration was higher (P < 0.05) in buck-kids than doelings (Table 6).

**Table 4:** Classification of the goats kids into the three tropical breeds during early and late neonatal life using discriminant analysis.

Neonatal period	Red Sokoto	Sahel	WAD	Total
Early (Days 0 and 3)				
Red Sokoto	62.50	33.30	4.20	100.00
Sahel	20.80	54.20	25.00	100.00
WAD	41.70	8.30	50.00	100.00
Late (Days 10 and 20)				
Red Sokoto	54.20	25.00	20.80	100.00
Sahel	16.70	58.30	25.00	100.00
WAD	25.00	20.80	54.20	100.00
Overall				
Red Sokoto	54.20	33.30	12.50	100.00
Sahel	18.80	54.20	27.10	100.00
WAD	37.50	12.50	50.00	100.00

**Table 5:** Structural matrix with discriminant functions for neonatal Red Sokoto, Sahel and WAD (West African Dwarf) goats.

Parameters	Function	
	1	2
Cholesterol	0.43*	-0.25
Triglyceride	0.41*	-0.11
Total protein	0.22	0.45*
Blood urea	-0.28	0.44*
Immunoglobulin	-0.35	-0.41*
Globulin	0.18	0.27*
Albumin	-0.11	0.20*

* Largest absolute correlation between each variable and any discriminant function

**Table 6:** Influence of sex on birth weight and serum metabolites in tropical goats during neonatal period.

Parameters	Doelings	Buck-kids	P value
Birth weight (g)	1.705 ± 0.12	1.950 ± 0.18	0.214
Total cholesterol (mg/dL)	137.90 ± 6.71	134.40 ± 7.03	0.717
Triglycerides (mg/dL)	71.07 ± 3.42	73.91 ± 4.19	0.599
Total proteins (g/L)	10.77 ± 0.26	10.58 ± 0.27	0.614
Albumin (g/L)	4.331 ± 0.16 a	4.921 ± 0.17 b	0.013
Blood urea (mg/dL)	46.42 ± 3.47	44.75 ± 2.31	0.680
Globulins (g/L)	6.68 ± 0.27 a	5.68 ± 0.29 b	0.014
Immunoglobulins (mg/mL)	29.24 ± 1.20	26.28 ± 1.48	0.119

Values with alphabets (a,b) indicates significant (P < 0.05) sexual dimorphism

## Discussion

Although the discriminant function classification shows some levels of metabolic homogeneity in the response of the three breeds to the novelty of neonatal life, the breed with hereditary dwarfism (WAD goat kids) [28] generally had higher metabolic status/parameters. The demonstration of an inverse relationship between birth weight and triglyceride concentration or the distinct association between dwarfism and triglyceride is to our knowledge novel in ruminants. Irrespective of gestational age and gender, a negative correlation has been consistently reported between birth weight and serum triglyceride concentration in humans [19, 20, 26, 29]. Similarly, a study in sheep also reported a progressive decrease in serum triglyceride concentration with an increase in birth weight [30]. Conversely in the latter study, serum total cholesterol concentration progressively increased with increase in birth weight. Although this is not in agreement with the current study, which demonstrated a negative association between birth weight and serum cholesterol concentration; both negative and positive correlations have been reported in humans [19, 20].

To our knowledge, the cause of the negative association between birth weight and serum triglyceride concentration is yet to be elucidated. It is possibly due to metabolic dependence of low birth weight neonates on lipid metabolites for cellular energy supply, due to small body mass that may lower the amount of glucose and amino acid stores in liver and muscles [4, 14, 30]. In accordance with the high serum triglyceride concentration in neonates with low birth weight, previous studies have reported lower glucose concentration in neonatal small ruminants with low birth weight [4, 30]. This birth weight-serum triglyceride interaction in neonatal small ruminants may be hormonally mediated by insulin as low serum glucose levels may reduce insulin production; thus reducing the suppressive effect of insulin on lipolysis, resulting in enhanced lipolysis and an increase in triglyceride levels [31, 32].

Similarly, variation in serum lipid based on birth weight in the three breeds explains the higher discriminant power of serum cholesterol and triglyceride in differentiating the response of the three breeds during neonatal life. Higher serum lipid in the dwarf goat kids may be associated with previously reported higher body and milk fats in the breed [33, 34]. The WAD goats seem to have a phenotypic feature that is consistent with disproportionate dwarfism, caused by signalling disruption which is either associated with hormonal or signalling-pathway abnormality [2, 35]. In dwarf Brahman cattle, a 40% reduction in growth hormone (GH) activity is evident, and an association between dwarfism and obesity has been reported in humans and rodents [17, 18]. GH-deficient children exhibit short stature and are mildly obese, and supplementation with GH diminished the adipose mass in these children [18, 36, 37]. In rodents, hypophysectomy or inactivation of a GH transgene caused obesity independent of feed consumption [17, 38]. Apparently, GH deficiency in mammals is associated with stunted somatic growth and increased adipose tissue, due to elimination of the lipolytic effect of GH in affected animals [18, 36]. Thus, we speculate that the high serum lipid profile in WAD goat kids in the current study may be due to dwarfism. There is paucity of data on body fat reserves in the three breeds, which could influence neonatal adaptation and circulating lipid profiles. Naturally, the WAD goats are chubbier and have higher body condition scores than the Red Sokoto and Sahel goats, even when exposed to the same management and nutritional conditions [28].

The low total cholesterol levels in Sahel goats as compared to other breeds in the current study agrees with the studies of Okonkwo et al. [39] and Njidda et al. [40] that indicated lower total cholesterol in Sahel than Red Sokoto and WAD goat kids. The low cholesterol and triglyceride levels in Sahel goat kids maybe a genetically-induced adaptive feature to enhance the adaptation of the breed to its hot Sahelian climate, as the low cholesterol levels, would be accompanied by low body fat to reduce heat production and enhance the efficiency of heat dissipation [17, 41].

According to O’Brien and Sherman [42], a total serum protein of less than 5.4 g/dL in neonatal goat kids is considered a failure of passive transfer of maternal immunity. On Day 0 in the current study, no Sahel and WAD goat kids had serum total proteins less than 5.4 g/dL, while some Red Sokoto goat kids had lower serum total proteins with one kid having a value (4.45 g/dL) less than 5.4 g/dL. This accounts for the left skewness in serum concentration of total proteins and immunoglobulins in the boxplot of Red Sokoto goats. On Day 3, however, all the affected kids had taken enough colostrum/milk to increase the serum total proteins to values above 5.4 g/dL. Immediately after suckling, the levels of all serum proteins begin to increase, mainly due to intestinal absorption of immunoglobulins and albumin [42, 43]. At about 15 days old, which correspond to the half-life of albumin in ruminants [44], the production of albumin by the liver and immunoglobulins by the B-lymphocytes would have commenced [45], resulting in a further increase in albumin and immunoglobulins, and by extension a rise in serum total proteins.

The lower concentrations of serum total protein and albumin in Red Sokoto than Sahel and WAD goat kids in the current study, agrees with the finding of Njidda et al. [40], which demonstrated lower concentration of albumin in Red Sokoto than Sahel goat kids. Nitrogen intake through dietary supply of amino acids is the most important requirement for albumin synthesis [46]. Since all breeds of goat in the current study were fed with the same diet, it is speculated that the low concentration of albumin in neonatal Red Sokoto goat kids may be due low intestinal uptake or may have been genetically influenced to reserve amino acids and redirect dietary nitrogen towards growth and development of body tissues. Furthermore, this may reflect a genetic variation in the mechanism through which the three breeds take-up and/or metabolize dietary nitrogen. We suggest the need for further studies to evaluate protein metabolism of these West African goats and speculate that the Red Sokoto goat kids may require high dietary protein supplementation through provision of concentrate feed for the kids and their does. Likewise, the higher albumin levels in male than female kids recorded in the current study conforms to the findings of Njidda et al. [40] in Red Sokoto goat kids. The higher serum albumin may serve as a reservoir of protein to support the fast growth rate in buck-kids [47] in after the neonatal period, as sexual in goat kids is mainly expressed after neonatal life [48].

Higher serum urea concentrations in Sahel and WAD goat kids, unlike in Red Sokoto goat kids, in the first 3 day of life, may reflect marked protein degradation and deamination of amino acids as well as high rate of tissue remodelling after birth [15]. The negative association between serum urea and birth weight suggests that birth weight could influence the protein catabolic adjustment in early neonatal life, with smaller breeds having greater need to catabolize protein, probably to satisfy gluconeogenic demands or remodel body tissues [14, 15].

Serum urea is strongly linked to supply, synthesis and degradation of proteins [48, 49] and previous studies have reported a higher serum urea concentration at birth with a subsequent decrease postnatally [50, 51]. Another perspective is that high serum urea in early neonatal life may reflect poor excretion rate and the reduction subsequently, may not be unconnected to enhanced elimination of urea through increased glomerular filtration rate and/or intestinal excretion as the neonates mature [49]. The lower concentration of serum urea in Red Sokoto goat kids further emphasises the earlier speculation that Red Sokoto goat kids showed some physiological adaptation aimed at economising dietary nitrogen and sparing available amino acids for growth and tissue development. This may explain the higher discriminant power of serum urea concentration (in the second discriminant function) in differentiating the neonatal adjustments of the three breeds, and also, justifies the low variability of serum urea concentration in Red Sokoto goat kids in the boxplot.

The Sahel goat breed which had the highest body weight, length and height as well as BMI during neonatal life seems to have a more efficient protein metabolism; indicated by the higher levels of serum albumin as well as the relatively higher serum total proteins and immunoglobulin at birth with no significant fluctuation after the increase at birth. However, the higher levels of serum urea may suggest that Sahel goats relied more on protein catabolism (as compared to other small size breeds) to compensate for the low the gluconeogenic energy supply due to low serum lipids in the Sahel goats. The gluconeogenic supply of energy is important in neonatal ruminants and needs to be compensated for, as it provides about 75% of their total glucose needs [9, 52]. This finding may infer a higher catabolism of serum proteins in the breed (Sahel goat) with low serum lipid, but higher body tissue mass, to complement energy demand.

The significant increase in the concentration of immunoglobulins on Day 3 in the three breeds of goat kids suggests an efficient intestinal uptake before gut closure [42, 43]. The subsequent progressive decrease in immunoglobulins after Day 3 in Red Sokoto and the dwarf kids is suggestive of clearance of passive immunity due to physiological degradation in blood, as there is insignificant or no endogenous production of immunoglobulins in the kids at the neonatal stage [45, 53]. Of the three breeds studied, neonatal Sahel goats showed a unique pattern in immunoglobulins concentration. Specifically, the concentration of immunoglobulins in Sahel goat kids was relatively higher at birth and remained relatively stable throughout neonatal life. This suggests an initial rapid period of immunoglobulin absorption in the intestine of Sahel goat kids. Positive association between immunoglobulin concentration in late neonatal life and birth weight shows the influence of birth weight on immunity and survival of tropical goats, postnatal. In ruminants, strong association exists between the successful transfer of passive immunity (TPI) and production performance; successful TPI is associated with long-term benefits such as improved body weight gain and feed efficiency [43, 54].

## Conclusion

A marked negative association exists between birth weight and serum triglyceride concentration during neonatal life in single-born neonatal goat kids. Dwarfism or small body size is associated with higher serum lipid profiles in goat kids. The Sahel goat kids which had larger body size at birth demonstrated lower lipid profiles, but higher profile of serum urea. Goat kids with higher birth weight may have elevated immunoglobulin concentration in late neonatal life. Thus, the birth weight could be useful in predicting the expected metabolic adjustments in neonatal tropical goat kids, and this information may serve as a valuable tool in manipulating maternal and neonatal diets to enhance survival. We recommend high dietary protein supplementation in Red Sokoto goat kids through provision of concentrate feed to the kids and their does.

## Supporting information

S1 Data (XLSX)
